# ZnO Nanoparticles/Reduced Graphene Oxide Bilayer Thin Films for Improved NH_3_-Sensing Performances at Room Temperature

**DOI:** 10.1186/s11671-016-1343-7

**Published:** 2016-03-08

**Authors:** Huiling Tai, Zhen Yuan, Weijian Zheng, Zongbiao Ye, Chunhua Liu, Xiaosong Du

**Affiliations:** State Key Laboratory of Electronic Thin Films and Integrated Devices, School of Optoelectronic Information, University of Electronic Science and Technology of China (UESTC), Chengdu, 610054 People’s Republic of China

**Keywords:** ZnO, Reduced graphene oxide (rGO), Bilayer, Thermal reduction, NH_3_, Heterojunction, 07.07.Df, 07.10.Cm

## Abstract

ZnO nanoparticles and graphene oxide (GO) thin film were deposited on gold interdigital electrodes (IDEs) in sequence via simple spraying process, which was further restored to ZnO/reduced graphene oxide (rGO) bilayer thin film by the thermal reduction treatment and employed for ammonia (NH_3_) detection at room temperature. rGO was identified by UV-vis absorption spectra and X-ray photoelectron spectroscope (XPS) analyses, and the adhesion between ZnO nanoparticles and rGO nanosheets might also be formed. The NH_3_-sensing performances of pure rGO film and ZnO/rGO bilayer films with different sprayed GO amounts were compared. The results showed that ZnO/rGO film sensors exhibited enhanced response properties, and the optimal GO amount of 1.5 ml was achieved. Furthermore, the optimal ZnO/rGO film sensor showed an excellent reversibility and fast response/recovery rate within the detection range of 10–50 ppm. Meanwhile, the sensor also displayed good repeatability and selectivity to NH_3_. However, the interference of water molecules on the prepared sensor is non-ignorable; some techniques should be researched to eliminate the effect of moisture in the further work. The remarkably enhanced NH_3_-sensing characteristics were speculated to be attributed to both the supporting role of ZnO nanoparticles film and accumulation heterojunction at the interface between ZnO and rGO. Thus, the proposed ZnO/rGO bilayer thin film sensor might give a promise for high-performance NH_3_-sensing applications.

## Background

As air pollutions become more and more serious, the demand for high-performance and low-cost gas sensors increases. Among the toxic gases of interest, ammonia (NH_3_) is one of the most harmful environmental pollutants with a strong and irritating smell aroused from different sources, such as combustion of chemical materials, electronic manufacturing, medical treatment, and ammonification by nitrogen cycle [1, 2]. Generally, an acceptable level of NH_3_ is 8-h exposure limit at 25 ppm and a short-term (15 min) exposure level at 35 ppm [3–5]. Therefore, there is a practical meaning to develop sensitive and reliable gas sensors operated at room temperature targeting NH_3_. Up to now, the most typical NH_3_-sensing materials are metal-oxides semiconductor systems (SnO_2_, ZnO, TiO_2_, WO_3_, In_2_O_3_, etc.). Among them, ZnO is one of the most promising materials due to its ideal chemical and thermal stability, low-cost and good gas-sensing responses [6, 7]. However, the high working temperature (200–500 °C), low selectivity, and slow response/recovery rate of metal-oxide-based sensors limit their further application. Thus, it still remains a great challenge to improve gas-sensing performances of present metal-oxide-based NH_3_ sensors, and further work should be performed.

Recent research has shown that composite of metal-oxide nanomaterials with other gas-sensing materials such as reduced graphene oxide (rGO) could enhance sensing properties of individual materials [7–13]. rGO is more beneficial for gas molecule detection compared with pure graphene due to its residual oxygen functional groups that provide an increased amount of adsorption sites [6, 14, 15]. Meanwhile, rGO has also great advantages of low-cost and bulk quantity production [7]. Up to now, different ZnO nanomaterials, such as nanoflowers [6, 9], nanoparticles [7, 16, 17], nanofibers [8], nanorods [11, 18, 19], and quantum dots [20, 21], were designed and used for ZnO/rGO gas-sensing hybrid materials preparation, and the enhanced sensing performances for various gases including nitrogen dioxide (NO_2_), hydrogen (H_2_), formaldehyde (HCHO), methane (CH_4_), and ethyl acetate have been reported and analyzed, which might be ascribed to more adsorption sites and creation of p-n heterojunctions, etc. [8–10]. However, despite their improved advantageous characteristics, these sensors still suffer from several shortcomings more or less, such as high operating temperature (more than 100 °C) [8, 11, 16–18], baseline drift [9], and long recovery time [16]. Therefore, further enhancement of gas-sensing properties is still strongly required.

Recently, in our lab, a novel TiO_2_/rGO layered film was proposed and prepared through simple thermal treatment for HCHO detection at room temperature, and an interesting abruption of rGO sheets during the heating process was focused and discussed in details [20]. Herein, a similar strategy was used for the development of ZnO/rGO bilayer thin film-based NH_3_ sensor operated at room temperature. The effect of rGO amount on sensing properties was investigated, sensing mechanism model was established, and interaction between NH_3_ molecules and sensor was further deciphered in the present work. The resultant bilayer film sensor might have great potential in the development of novel NH_3_ gas sensor.

## Methods

### Sensor Fabrication

**Fig. 1:**
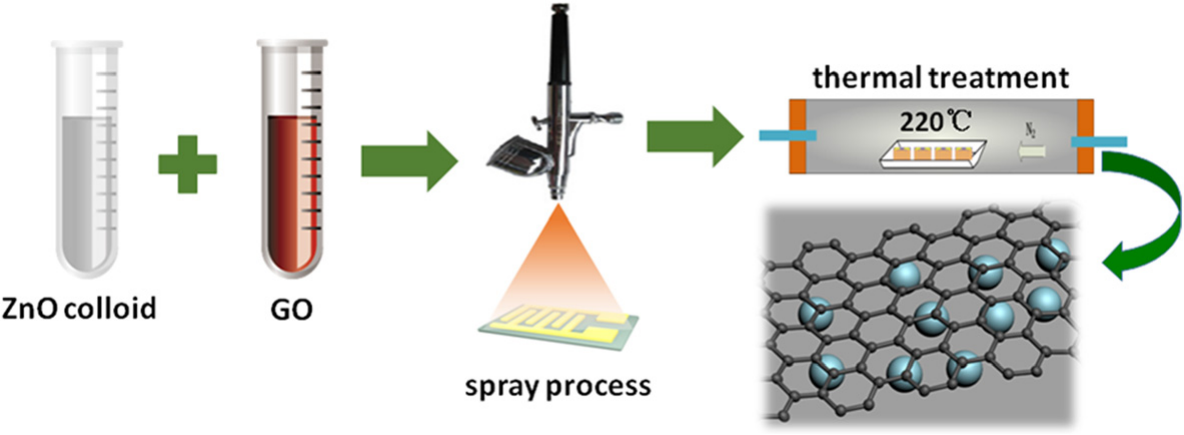
Schematic diagram shows that ZnO nanoparticles colloid and different amount GO solutions were sprayed on IDEs in sequence, and then ZnO/GO bilayer films were further restored to ZnO/rGO by thermal reduction process

Similar details of experimental process on metal-oxide nanoparticles/rGO-based bilayer thin film were reported in our earlier work [22]. GO was synthesized from natural graphite flakes through Hummer’s method. In a typical process run, as shown in Fig. 1, firstly, 2 mg/ml ZnO colloid in ethanol (purchased from Sigma-Aldrich) and 0.5 mg/ml GO aqueous solution were obtained through dilution and then sonicated, and secondly, 1 ml ZnO colloid and different amount GO solution (0.5, 1, 1.5, 2, and 2.5 ml) were deposited on gold interdigital electrodes (IDEs) in sequence by spraying process. After that, ZnO/GO bilayer thin film samples were restored to ZnO/rGO films through thermal reduction process (in nitrogen flow, 220 °C for 2 h). The corresponding sample was noted as ZnO/rGO-0.5, ZnO/rGO-1, ZnO/rGO-1.5, ZnO/rGO-2, and ZnO/rGO-2.5, respectively. Pure rGO (1 ml) film was also prepared under the same condition for comparison.

### Characterization and Measurement

The surface morphology features of prepared thin films were observed using a field emission scanning electron microscope (FESEM, S-4800, Hitachi Ltd., Japan). Ultraviolet-visible (UV-vis) spectra were measured with UV-1700 pharmaSpec (Japan, Shimadzu) in the range of 190–800 nm. The X-ray photoelectron spectroscope (XPS) analysis was performed with a commercial X-ray photoelectron spectrometer (Scienta ESCA-200) using MgKα X-ray source.

The electrical properties of sensors were measured by Keithley 4200-SCS. For gas-sensing properties evaluation, the fabricated sensors were placed in a homemade gas sensor assembly (Teflon chamber), and resistance changes of sensors were recorded at room temperature by a Keithley 2700 data acquisition system connected to a computer. Mass flow controllers (MFC, MT50-4J, Beijing Metron Instruments Co. Ltd, China) were employed to vary the concentration of standard NH_3_ vapors (100 ppm) in dry air (carrier gas). The tested chamber was purged with dry air between each NH_3_ pulse to allow the film surface to recover. The sensor response (*R*) is defined as *R*% = (*R*_gas_ − *R*_air_)/*R*_air_ × 100, where *R*_gas_ and *R*_air_ are the resistance in tested gas and dry air, respectively. The response/recovery time is defined as the sensor achieved 90 % of total resistance change.

## Results and Discussion

### Characterization

**Fig. 2:**
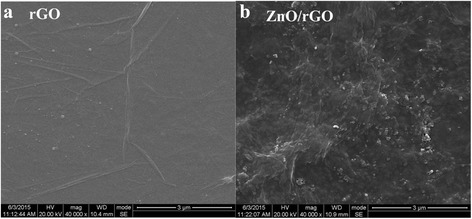
Representative FESEM images of **a** rGO and **b** ZnO/rGO-1 films. The surface morphology features of ZnO/rGO bilayer film demonstrates obvious fluctuant transparent rGO nanosheets with dispersed ZnO nanoparticles or their aggregation

Representative FESEM micrographs of single rGO and ZnO/rGO-1 film are shown in Fig. 2. Figure 2a represents rGO nanosheets with slimsy wrinkles whereas Fig. 2b displays obvious fluctuant transparent rGO nanosheets with dispersed ZnO nanoparticles or their aggregation, which should be ascribed to the supporting function of ZnO nanoparticles under folded rGO sheets. It is conjectured that ZnO/rGO bilayer film should possess larger surface area than pure rGO one, and good adhesion between rGO and ZnO nanoparticles might also be formed with the aid of thermal reduction treatment [17].

**Fig. 3:**
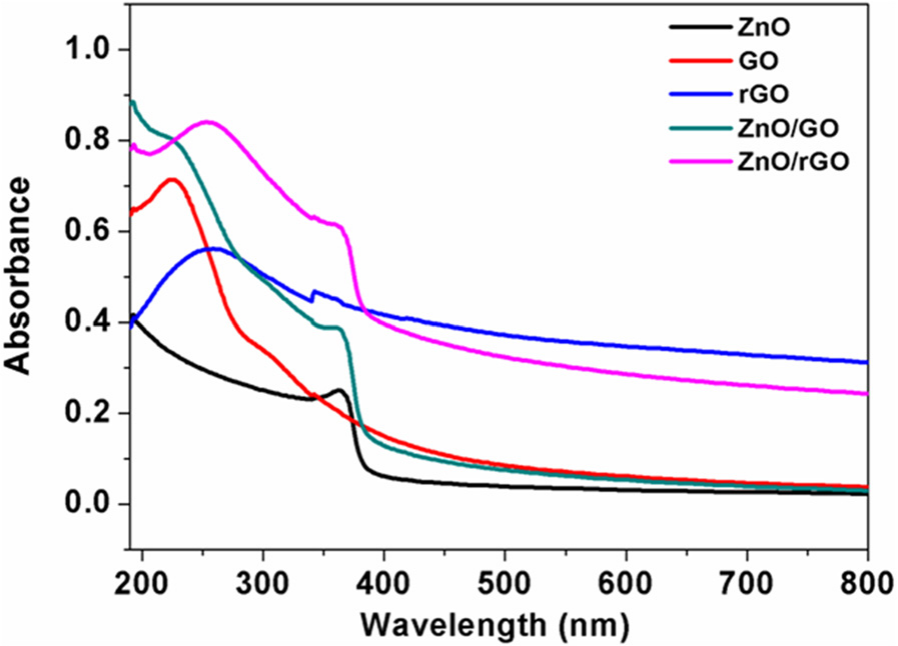
The UV-vis spectra indicates that GO has been restored to rGO through thermal treatment, and ZnO/rGO bilayer film is composed of ZnO and rGO components

**Fig. 4:**
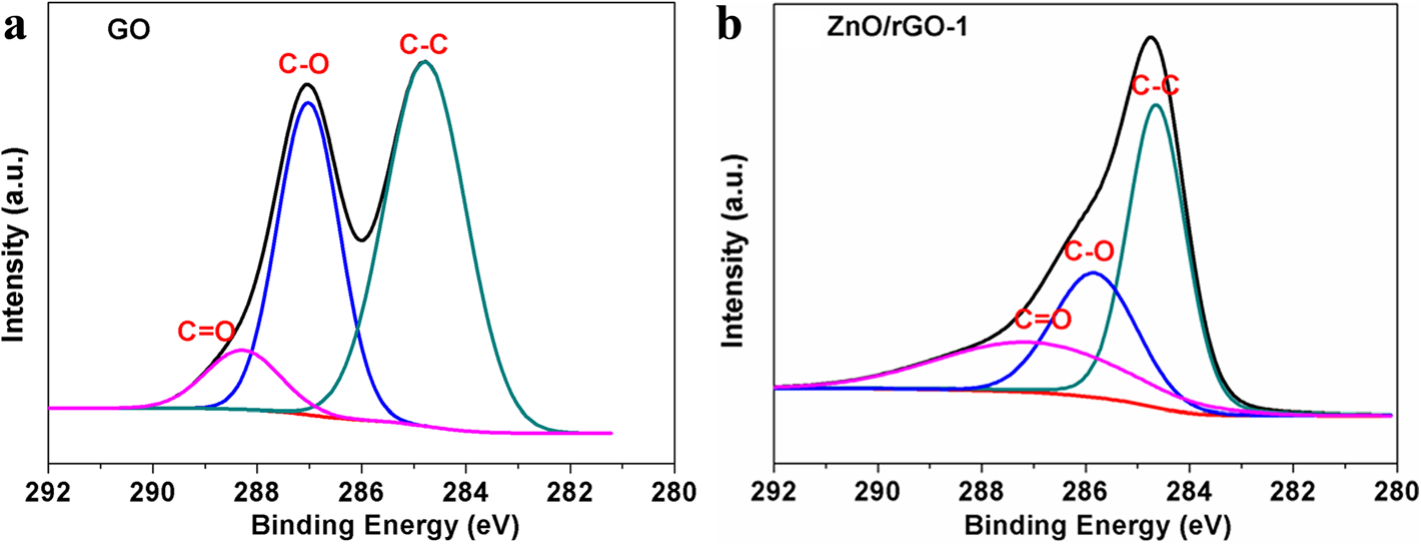
High-resolution C1s XPS spectra of **a** GO and **b** ZnO/rGO-1 films. The XPS analysis further confirm the presence of rGO in ZnO/rGO thin film after thermal reduction process

Figure 3 shows UV-vis absorption spectra of prepared different films. In the absorption spectrum of ZnO/GO film, it shows both absorption peaks of GO at ca. 226 nm (π-π* transitions of C–C bonds) [23] and ZnO at ca. 362 nm (the intrinsic band gap absorption) [24], referred to pure ZnO and GO films shown in Fig. 3. The spectrum of ZnO/rGO-1 bilayer thin film also exhibits the overlapping absorption bands of ZnO and rGO (ca. 254 nm, the excitation of π-plasmon of the graphitic structure). However, the ZnO/rGO film showed a slight blue shift from 366 to 361 nm compared with pure ZnO, and from 259 to 252 nm compared with pure rGO, which might be explained by the quantum size effect of the fine structure in nanometer regime [23]. It has been proved that the excitation energy of ZnO nanoparticles will increase with the decrease of grain diameter according to the Kubo theory [25]; therefore, the blue shift of ZnO absorption peak in ZnO/GO-layered film might demonstrate the decrease of ZnO nanoparticles size and further indicate certain synergistic effect between ZnO and rGO layers. Meanwhile, ZnO/rGO film exhibits an enhanced optical absorption in the UV-visible spectral region, which was also observed and reported in ref. [18]. The reduction of GO was further investigated by XPS spectra of C elements present in GO and ZnO/rGO-1 samples, as shown in Fig. 4a, b. It can been seen that C1s spectra of both samples could be deconvoluted into three Gaussian peaks associated with C–C, C–O, and C=O, respectively. Compared with the peak intensity of C–O and C=O in GO (Fig. 4a), the peak intensities of C–O and C=O in ZnO/rGO sample decrease obviously, indicating that GO has been restored to rGO successfully with some amount of residual oxygenic groups after thermal reduction [7, 16, 24, 26], which is also consistent with UV-vis spectra results.

### Electrical and Gas-Sensing Properties

**Fig. 5:**
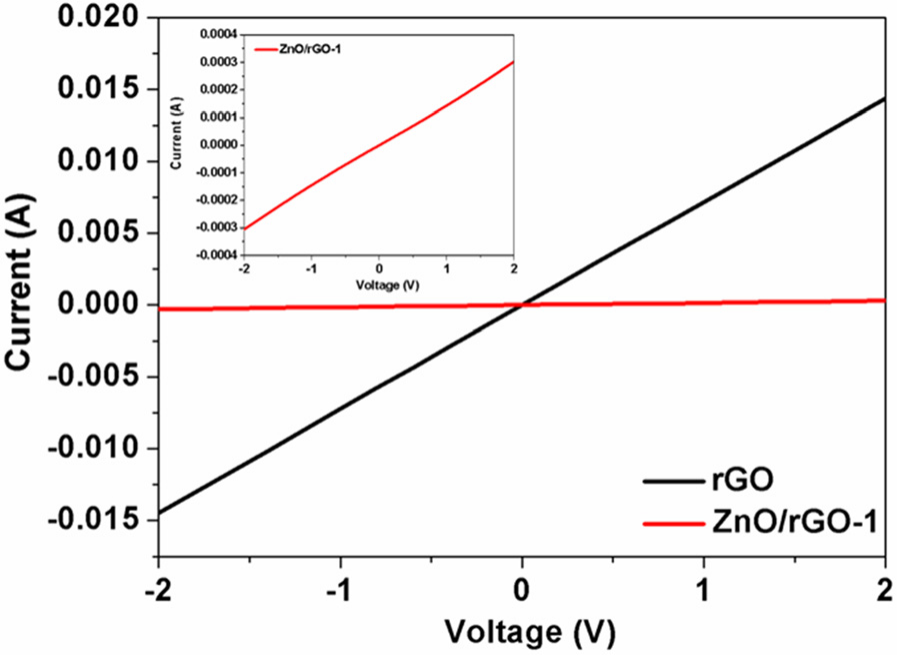
rGO and ZnO/rGO samples are the typical p-type semiconductor, and the resistance of rGO increases after the introduction of lower ZnO nanoparticles layer

The electrical behavior of rGO and ZnO/rGO-1 film samples was investigated at room temperature, and the corresponding current-voltage (*I*-*V*) curves were displayed in Fig. 5, in which the magnified *I*-*V* curve of ZnO/rGO-1 sample was inset. Both samples exhibit the typical p-type semiconductor and almost linear characteristics, indicating the ohmic nature of films. It is noteworthy that the resistance of ZnO/rGO bilayer thin film is two orders higher than that of rGO, and the reason for this phenomenon might be that ZnO usually has a much higher resistance at room temperature compared to rGO; meanwhile, some discontinuous ZnO nanoparticles under rGO layer are also unhelpful for charge transport in ZnO/rGO bilayer thin film.

**Fig. 6:**
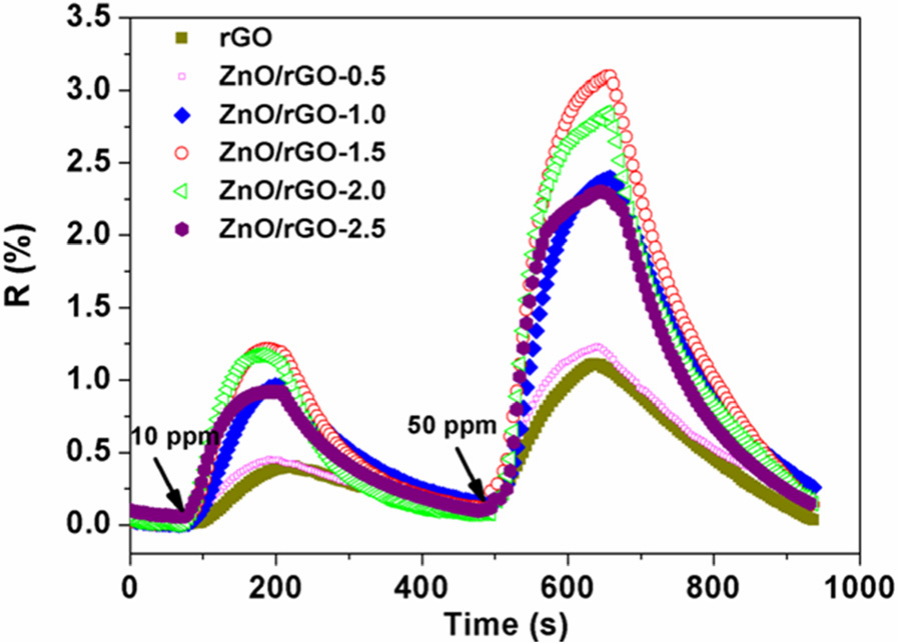
The enhanced sensing performances of bilayer thin film sensor are observed clearly compared with pure rGO sensor, which also are influenced by the sprayed GO amounts. The optimal GO amount is 1.5 ml

The response-recovery behavior is one of the important properties for evaluating the characteristics of gas sensors; therefore, dynamic response curves of pristine rGO and different ZnO/rGO bilayer thin film sensors are shown in Fig. 6 when exposed to 10 and 50 ppm NH_3_ at room temperature. Apparently, the resistances of all sensors show an increased trend when adsorbing NH_3_ vapor (reducing gas), further indicating the p-type semiconductor characteristic of rGO and ZnO/rGO samples. The response values, response, and recovery times of all samples were calculated and summarized in Table 1. Obviously, ZnO/rGO bilayer thin film sensors all exhibit overall improvement including larger response values and shorter response/recovery time than those of bare rGO one, and ZnO/rGO-1.5 sample shows the highest response, almost three times higher than pure rGO one. It is also noticeable that the response values of ZnO/rGO samples increase with increasing rGO amount and then decrease with a further rise of rGO amount. The maximum response value and shorter response/recovery time were obtained when sprayed GO amount was 1.5 ml. On the basis of these results, ZnO/rGO-1.5 sample was chosen for the subsequent investigations.

**Table 1:** The response values and response/recovery times of all samples when exposed to 10 and 50 ppm NH_3_. The results show that ZnO/rGO bilayer thin film sensors exhibit superior sensing properties than bare rGO one, and the optimal GO amount is 1.5 ml

Sensors	Response value (%)		Response time (s)		Recovery time (s)	
	10 ppm	50 ppm	10 ppm	50 ppm	10 ppm	50 ppm
rGO	0.38	1.08	108	92	278	268
ZnO/rGO-0.5	0.44	1.22	102	86	274	256
ZnO/rGO-1.0	0.95	2.38	88	82	208	219
ZnO/rGO-1.5	1.20	3.05	78	84	188	216
ZnO/rGO-2.0	1.16	2.81	80	75	160	212
ZnO/rGO-2.5	0.92	2.30	82	68	183	223

**Fig. 7:**
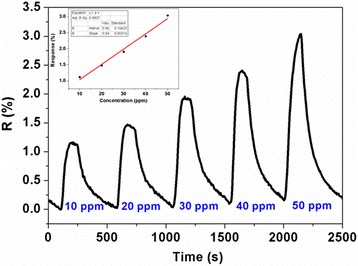
The results exhibit the good reversibility and linear characteristic of the sensor

**Fig. 8:**
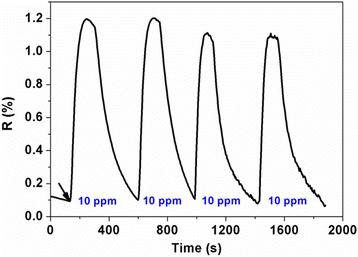
A good reproducibility with less than 10 % decrease in response could be obtained

**Fig. 9:**
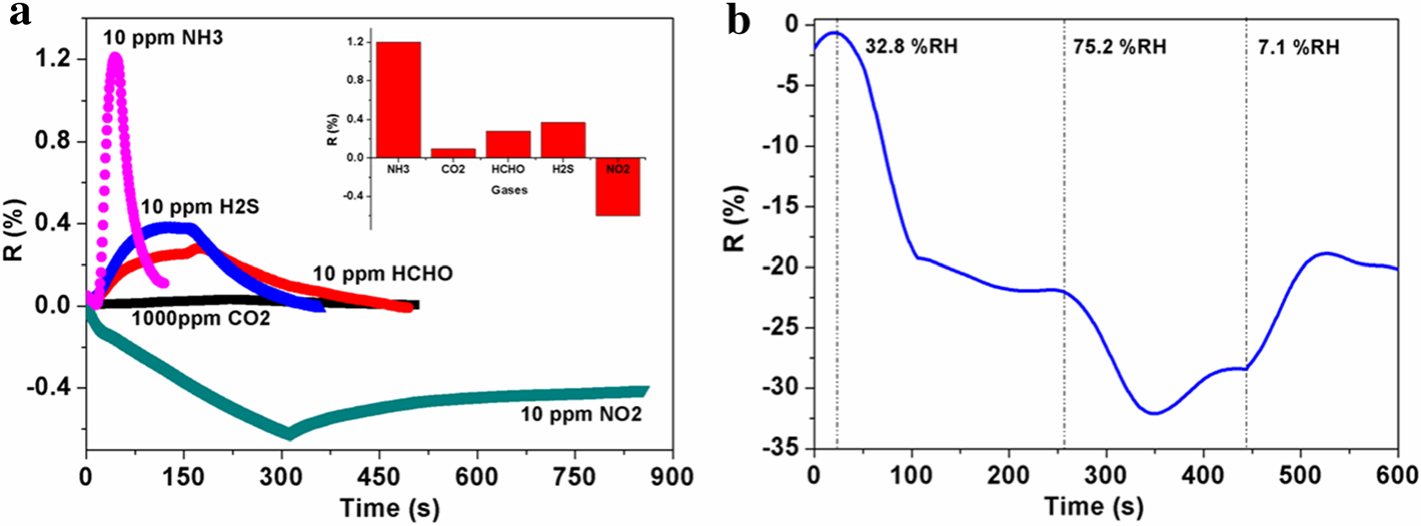
**a** Compared with four kinds of interfering gases, the greater response values and shorter response/recovery times of the sensor to NH_3_ were observed obviously, exhibiting the good selective ability to NH_3_. **b** The response of the sensor to water molecules is much larger (one or two orders of magnitude) than that of the sensor when exposed to NH_3_ or other tested interfering gases, indicating that the interference of moisture on the prepared sensor is non-ignorable

The real-time response curves of ZnO/rGO-1.5 sensor are plotted in Fig. 7 when exposed to a series of NH_3_ concentration ranging from 10 to 50 ppm at room temperature. It shows that the response increases quickly upon exposure to NH_3_ and returns to its initial baseline after exposure to dry air, suggesting the good reversibility of optimized sensor in NH_3_ sensing. The response and recovery time were found to be within 1.5 and 4.0 min, respectively. Furthermore, the relationship of response values as a function of NH_3_ concentration and its fitting curve has also been investigated and inset in Fig. 7, showing a good linear characteristic of responses versus NH_3_ concentration with a regression coefficient (*R*^2^) of 0.9827. The reproducibility of the sensor was also assessed for sequential four pulses of 10 ppm NH_3_, and the result is exhibited in Fig. 8, indicating good reproducibility with less than 10 % drift in response. Furthermore, to evaluate the selectivity of the sensor, ZnO/rGO-1.5 sensor was exposed to different interfering gases including carbon dioxide (CO_2_, 1000 ppm), HCHO (10 ppm), NO_2_ (10 ppm), and hydrogen sulfide (H_2_S, 10 ppm), and the transient gas-sensing response curves are shown in Fig. 9a, in which the response values versus NH_3_ and interfering gases are inset. As expected, the response value of ZnO/rGO-1.5 sensor towards 10 ppm NH_3_ presents about 2–30 times greater than that to other tested gases; meanwhile, it is obvious that response/recovery rates of sensor to NH_3_ are significantly quicker than those to other gases. Consequently, the sensor exhibited superior selective ability to NH_3_. Compared with some recent reports in ZnO [25, 27, 28] or graphene [29, 30] based NH_3_ sensors, the as-prepared ZnO/rGO thin film sensor in our work exhibits lower cost, better reversibility, smaller detection concentration, and quicker response/recovery rates. Meanwhile, the spraying process is also simple and feasible. However, it was found in the experiment that the resistance of prepared ZnO/rGO film sensor decreased promptly with exposure to 32.8 % RH and 75.2 % RH (7.1 % RH level was served as the baseline) and then gradually reached a relatively stable response value (ca. 20 and 32, respectively) accompanied with an incomplete recovery, as shown in Fig. 9b. It is obviously seen that the response of the sensor to water molecules is much larger (one or two orders of magnitude) than that of the sensor when exposed to NH_3_ or other tested interfering gases, indicating that the interference of water molecules on the prepared sensor is non-ignorable. The mechanism of this phenomenon should be ascribed to the residual oxygenic groups (high hydrophilicity) of rGO [31] and active sites (oxygen vacancies) of ZnO for dissociation of water molecules [32]. Therefore, some proper techniques should be researched to eliminate the effect of moisture in the further work.

### Sensing Mechanism

**Fig. 10:**
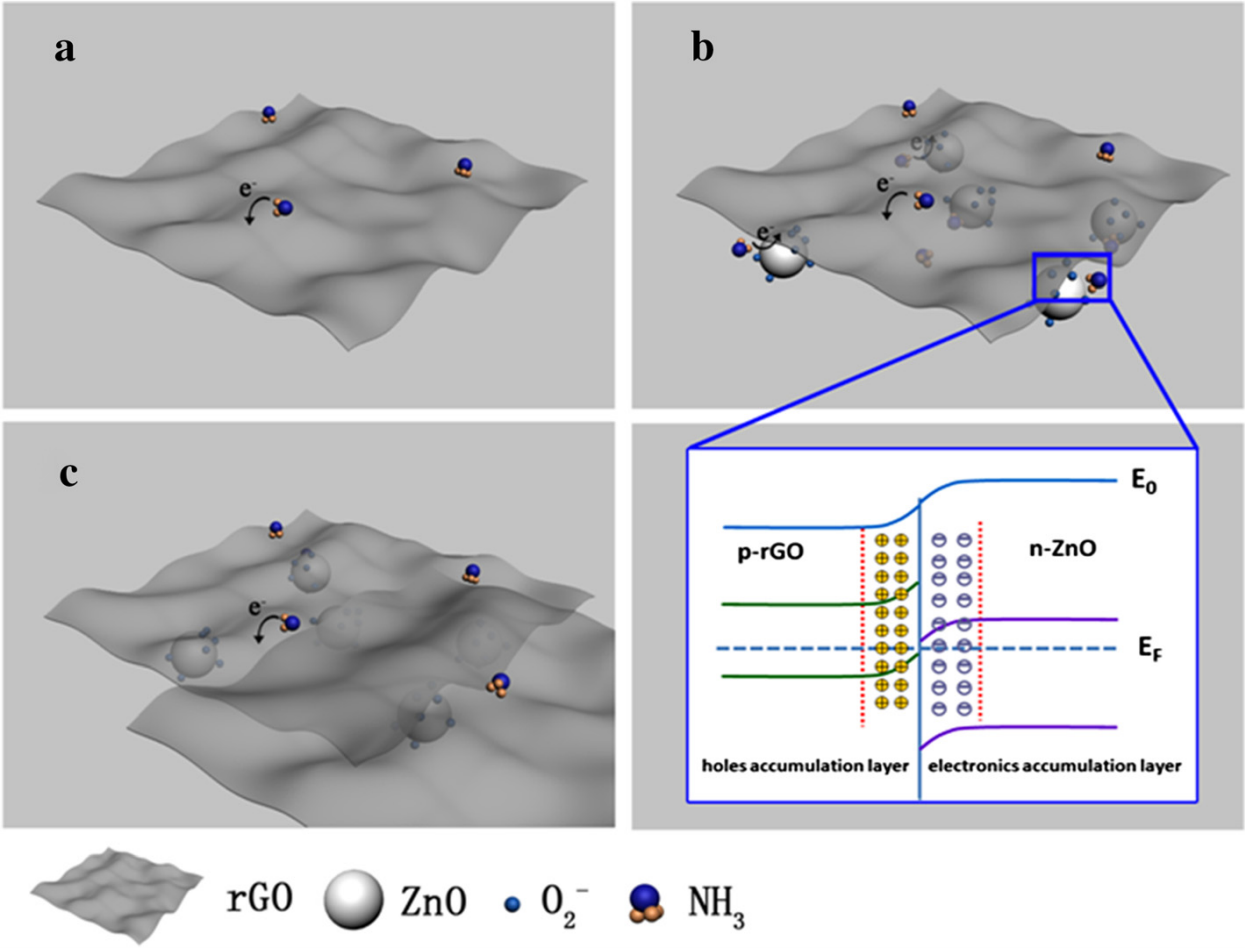
Schematic illustration of NH_3_-sensing mechanism of (**a**) rGO and (**b**)~(**c**) ZnO/rGO film sensor, in which (**b**) and (**c**) shows the adsorption sites of ZnO/rGO film with proper and excess rGO amount, respectively, and the magnified view in (**b**) is the formed accumulation heterojunctions at the interface

Many researchers have reported improvement in gas-sensing properties due to heterojunction formation between metal oxide and rGO for their hybrids [7–11, 16, 18–21]. Meanwhile, for metal-oxide nanoparticles/rGO-layered thin film sensor, a preliminary sensing mechanism has also been discussed in our previous report [22], in which the supporting and catalytical roles of lower TiO_2_ nanoparticles were proposed. Based on the above reports, the enhanced NH_3_-sensing performances of ZnO/GO film sensor at room temperature are proposed in this present work, which should be ascribed to two aspects, one is the supporting role of ZnO nanoparticles layer (as illustrated in ref 22) and the other is the formed accumulation heterojunction at interface between rGO and ZnO. Furthermore, the effect of upper rGO amount on sensing properties is also focused. A schematic illustration of sensing mechanism is shown in Fig. 10.

For bare rGO layer, as shown in Fig. 10a, the electrons transfer from reducing NH_3_ gas molecules to p-type rGO nanosheets and result in a decrease of charge carriers concentration (the resistance increases). After an appropriate amount of rGO nanosheets are deposited on the surface of ZnO nanoparticles film, we suggest two kinds of adsorption sites, being related to rGO nanosheets surface and ZnO/rGO interfaces, exist in bilayer thin film at room temperature, in which the latter one might be responsible for the enhancement of sensing properties compared with pure rGO film. It is noteworthy that although O^2−^ could be produced on the surface of ZnO nanoparticles even at room temperature (see Fig. 10b), the catalytic oxidation ability of O^2−^ is not powerul enough to convert NH_3_ as the final product (N_2_ and H_2_O) when the temperature is lower than 373 K [27]. However, since the work function of p-rGO (4.75 eV) is lower than that of n-ZnO (5.20 eV), a considerable amount of local accumulation heterojunctions at interfaces between rGO nanosheets and ZnO nanoparticles might be formed at ZnO/rGO interfaces [8], in which more holes and electronics could accumulate in rGO and ZnO regions of heterojunction, respectively, as shown in the magnified view of Fig. 10b. In the present case, rGO becomes more p-type than the original state, and thus, the energy barrier between absorbed NH_3_ molecules and rGO is further reduced [11], resulting in the higher response values and shorter response times. However, in case of ZnO/rGO-2.0 and ZnO/rGO-2.5 samples, it is possible that an excess of rGO might weaken the supporting role of lower ZnO nanoparticles film and further reduce the probability of target NH_3_ gas molecules adsorption at heterointerfaces between ZnO and rGO films, leading to a decline in sensor responses, as shown in Fig. 10c.

## Conclusions

In summary, ZnO/rGO bilayer sensitive thin film was produced through a two-step process. In the first step, ZnO nanoparticle film and GO layer were sprayed on gold interdigital electrodes in sequence, and then GO was successfully restored to rGO by the thermal reduction method at 220 °C for 2 h, as proved by the UV-vis spectra and XPS analyses. The experimental results showed that ZnO/rGO bilayer sensing film exhibited a comprehensive enhancement of room-temperature NH_3_-sensing performances compared with pure rGO one, including higher response values (~threefold better than that of rGO) and shorter response/recovery time (ca. 30/90 s was shorten). Meanwhile, the sensing properties were influenced significantly by the sprayed rGO amount, and the optimized amount of rGO was found to be 1.5 ml. The reason for the sensing property improvement was interpreted from the views of supporting roles of ZnO nanoparticle layer and accumulation heterojunctions at ZnO/rGO interfaces. Furthermore, the optimal sensor possessed short response time (<1.5 min), excellent linear characteristic, reversibility, reproducibility, and selectivity upon exposure to NH_3_ at room temperature. This sensor shows great potential for applications as room temperature operated NH_3_ gas sensor.

## Abbreviations

IDEs: interdigital electrodes
rGO: reduced graphene oxide
